# “Rusticall chymistry”: Alchemy, saltpeter projects, and experimental fertilizers in seventeenth-century English agriculture

**DOI:** 10.1177/00732753211033159

**Published:** 2021-09-17

**Authors:** Justin Niermeier-Dohoney

**Affiliations:** University of Chicago, USA

**Keywords:** Alchemy, vitalism, saltpeter, artificial fertilizers, British Agricultural Revolution, Hartlib Circle

## Abstract

As the primary ingredient in gunpowder, saltpeter was an extraordinarily important commodity in the early modern world. Historians of science and technology have long studied its military applications but have rarely focused on its uses outside of warfare. Due to its potential effectiveness as a fertilizer, saltpeter was also an integral component of experimental agricultural reform movements in the early modern period and particularly in seventeenth-century England. This became possible for several reasons: the creation of a thriving domestic saltpeter production industry in the second half of the sixteenth century; the development of vitalist alchemical theories that sought a unified explanation for the “growth” of minerals, metals, and plants; the rise of experimental natural philosophy; and the mid-seventeenth-century dominance of the English East India Company in the saltpeter trade, which allowed agricultural reformers to repurpose domestically produced saltpeter in agriculturally productive ways. This paper argues that the Hartlib Circle – a loose network of natural philosophers and social reformers – adopted vitalist matter theories and the practical, experimental techniques of alchemists to transform agriculture into a more productive enterprise. Though their grandiose plans never came to fruition, their experimental trials to develop artificial fertilizers played an early role in the origins and development of saline chemistry, agronomy, and the British Agricultural Revolution.

## Introduction

Writing about saltpeter in 1653, alchemist and agriculturist Cheney Culpeper made note of the troubling fact that “the Matter by w*hi*ch men are killed & fedde, is but one & the same, & differs onely in the minde & hande that uses it.”^1^ Culpeper was referring to the disturbing paradox that saltpeter appeared to be both a source of destruction – as the principal ingredient in gunpowder – and a wellspring of life for its capacity to fertilize crops. It seemed responsible for both life and death, growth and devastation. This dichotomy perplexed many early moderns. Peter Whitehorne, whose 1562 book provided the first English account of its chemical properties and manufacturing instructions, epitomized sixteenth-century attitudes toward saltpeter when he wrote that he could not “tell how to be resolved what thing it properly is” for “it seemeth it hath the sovereignty and quality of every element.”^2^ William Shakespeare wrote of the “great pity” that “villainous saltpetre should be digged out of the bowels of the harmless earth.”^3^ Unable to define it, the English physician William Clarke simply referred to it as the “hermaphroditical salt.”^4^

This complexity lent itself to versatility, and people throughout the early modern world applied it in an amazing variety of ways. German and English sources mentioned it as a food preservative, a meat tenderizer, and a beverage cooler for beer. Pharmacological sources touted it as a remedy for sensitive teeth, a treatment for breathing problems, and a cure for numerous ailments including skin lesions, itchiness, inflamed testicles, cholic, gout, and fistulas. Mixed with butter or other fats, it waterproofed leather and cleaned linen. Glassmakers, dyers, and engravers employed it in their trades. Some even believed it suppressed sexual desire in men.^5^

However, by far the most important use for saltpeter was in gunpowder, and procuring it had become an issue of state security by the fifteenth century, when it became a mainstay in European armies. Because of this, much of the attention historians have paid to saltpeter has understandably focused on its military applications and its value as a commodity in international trade and imperial expansion. David Cressy’s recent book-length treatment of the subject has illuminated the complicated political, social, and legal entanglements related to securing supplies of it and the role it played in both the centralization of the English state and the later expansion of the British Empire.^6^ On that latter point, James W. Frey has demonstrated that the rise of Britain as a global imperial power in the eighteenth century and the military revolution that made this possible owed a great deal to its success in cornering the market on Indian saltpeter.^7^ By replicating or “reworking” seventeenth-century procedures to make saltpeter, Haileigh Robertson and members of the Medieval Gunpowder Research Group at the University of Leeds have revealed the extraordinary challenges craftsmen faced and the razor-thin margins of error they confronted.^8^ Saltpeter has factored into numerous histories of firearms, military technology, and Renaissance warfare.^9^ Though these works have all shed a great deal of light on how early moderns engaged with saltpeter in its most important material role, far less attention has been paid to its non-military applications. Histories of saltpeter as an integral component of agricultural improvement, in early agronomic attempts to develop artificial fertilizers, and in early modern botanical experimentation are particularly lacking.^10^

England in the mid seventeenth century is an ideal location for such inquiries. By then, several related factors intersected to allow wider agricultural implementation and more novel and in-depth chemical and alchemical experimentation with saltpeter fertilizers to flourish. First, over the previous century, England had developed a thriving domestic saltpeter production industry, and while it began to decline in the middle of the seventeenth century due to far cheaper imports from India, much of the infrastructure and technical expertise remained intact.^11^ Second, beginning in the late fifteenth century, alchemists began rapidly integrating vitalist interpretations of matter into their theories of metallic and mineral generation, arguing that an animating force pervaded the cosmos and contributed to the inception, growth, and development of mineral, plant, and animal matter.^12^ This suggested analogs in botanical and biological domains. Attempts to define and isolate the vitalizing properties of matter abounded in these new natural philosophical contexts. Sixteenth-century alchemists like the iconoclastic Theophrastus von Honheim, better known as Paracelsus, expanded upon these new theories and practices and helped transform alchemy from a somewhat peripheral branch of mineralogy and metallurgy to a comprehensive, interpretative matter theory at the center of early modern European intellectual culture with bearings on botany, horticulture, and agriculture.^13^ Under these auspices, experimental methodologies inspired by artisans and craftsmen, and popularized by Francis Bacon, Bernard Palissy, and Hugh Plat, among others, became more common practical tools for investigating natural phenomena. Natural philosophers and skilled craftsmen alike made major advances in saline chemistry, which contributed to a much greater understanding of saltpeter’s potential function in everything from soil fertility and seed germination to plant respiration and the composition of human blood.^14^ Third, the broader group of natural philosophers, agricultural reformers, and practical experimenters who set to work applying these diverse bodies of saltpeter knowledge toward agricultural ends in England shared certain utopian social ideals and cornucopian economic objectives.^15^ They believed that there were no serious ecological, economic, or environmental problems that did not have technical or scientific solutions and that through the proper investigation of nature, human welfare could be enhanced and society improved.^16^ The Hartlib Circle – a sprawling correspondence network of scholars and social reformers centered around the Prussian émigré to England Samuel Hartlib – spearheaded many attempts to artificially produce saltpeter for agriculture with these goals in mind.^17^

My goal here is to demonstrate that mid-seventeenth-century English agricultural reformers developed a sophisticated experimental chemical philosophy designed to create or repurpose extant saltpeter in agriculturally productive ways. Members of the Hartlib Circle were enthusiastic about all manner of schemes and projects devoted to improving social welfare, relieving poverty, increasing public revenues, and reforming political structures, and agricultural projects were only one category of many.^18^ For them and their successors involved in agricultural improvement in the later seventeenth century, saltpeter appeared to be the most promising material substance to probe the secrets of botanical growth. For this work, members of the Hartlib Circle drew upon a wide range of sources including husbandry manuals, pamphlets on agricultural improvement, metallurgy and mineralogy texts, manuscripts of alchemical recipes, and the published works of practicing alchemists. Vitalist alchemists and laborers in the saltpeter industry regularly exchanged knowledge on these topics. Agricultural reformers took advantage of this, and the Hartlib Circle’s experimental trials to make artificial chemical fertilizers stood at the center of this knowledge exchange. Through what seventeenth-century gentleman farmer Peter Smith referred to as “rusticall chymistry,” these reformers attempted to artificially enhance soil fertility, improve crop yields, and provide long-term social stability through artificially augmented agricultural abundance.^19^

## Early modern saltpeter knowledge: Mining, manufacturing, and alchemy

Saltpeter has a long history in the West. Though first unambiguously mentioned in a late fifth-century Chinese text and described in Indian, Persian, and Arabic sources during the Middle Ages, the earliest Latin text containing a description of saltpeter and its combustible properties dates to the early thirteenth century and is itself most likely a translation of an earlier Arabic text.^20^ Roger Bacon, Albertus Magnus, Michael Scot, and Marcus Graecus all mentioned saltpeter as a component of gunpowder in the mid thirteenth century, though none described how to source it.^21^ Before modern times, the term “saltpeter” – now used exclusively to refer to potassium nitrate – was sometimes used interchangeably with niter and *nitrum*, making it difficult for historians to discern precisely what compounds are being discussed in historical documents. Herodotus, Pliny the Elder, Strabo, and the Old Testament books of Proverbs and Jeremiah all mention niter, though modern scholarship on these references suggests that these allusions probably refer to soda or sodium carbonate rather than potassium nitrate.^22^ By the time later medieval Latin writers described it, its incendiary and propulsive properties helped distinguish it from other salts.

Biochemically speaking, saltpeter occurs naturally when bacteria consume animal dung, urine, or other decomposing organic matter, producing urea that other species of bacteria then oxidize into ammonia, then nitrites, and finally nitrates.^23^ In locations with dense vegetation, this forms a potent fertilizer. In arid and semi-arid locations like deserts – but also notably in places where vegetation is grazed by livestock – these nitrates eventually combine with various carbonates, including potassium carbonate. Over time, the nitrates replace the carbonates and form potassium nitrate.^24^ Only a handful of places around the world possess the right climatic conditions to produce saltpeter naturally in abundance, which requires seasonal humidity followed by seasonal aridity. By the sixteenth century, well-known locations included Egypt; southeastern Spain; the Mediterranean coasts of North Africa and the Middle East; Hubei, Hunan, and Shandong in northeastern China; and Bihar and Bengal in northeastern India.^25^

Due to the lack of naturally occurring saltpeter throughout much of the continent, most European nations had three choices for securing stable supplies for their burgeoning gunpowder industries: mine it from the scant domestic sources available; import it from foreign nations; or create artificial manufacturing centers.^26^ The majority developed a three-pronged approach to take advantage of all of these sources. However, given the poor environmental conditions and capriciousness of the international market, many relied heavily on manufacture and resorted to the creation of saltpeter plants, sometimes called plantations or nitraries. Here, laborers processed nitrous earth – soil laden with decomposing organic matter – over months or years, after which they leached the mixture with water and wood ash, boiled the solution for long periods at high heat, and extracted the crystallized saltpeter, relying on techniques honed over decades through trial and error.^27^ Saltpetermen roved the countryside with royal license to dig and remove nitrous earth in stables, barnyards, gutters, latrines, outhouses, chicken coops, and dovecotes. If this material could not be obtained, saltpetermen created it themselves on-site at nitraries, where they “planted” beds of manure, urine, and compost and tended them for months until the soil was ready to be processed.^28^ These methods emerged in China in the Middle Ages, diffused into India, Persia, and the Ottoman Empire, and, by the early fifteenth century, spread to Europe thanks to Venetian merchants who came into contact with them in the eastern Mediterranean.^29^ There is some evidence to suggest that elite administrators of these projects, as well as skilled laborers, read and wrote texts describing how to distill, crystallize, and synthesize salts and other saline products based on these experiences. Evidence also suggests that alchemists drew on information gleaned from these practical efforts to substantiate their matter theories. Although the sixteenth century saw much of this information committed to text, this was a makers’ knowledge, developed over centuries, sometimes guarded along with other trade secrets, and usually passed along orally from master to apprentice.

The knowledge of saltpeter production in England during the early sixteenth century came almost entirely from continental saltpetermen hired by the English crown and the plagiarized writings of continental alchemists, metallurgists, and salt workers. As early as the 1510s, the English began supplementing their gunpowder supply with foreign imports to make up for a dearth of domestic mines. The Ordnance Office under Henry VIII employed German and Italian gunners and engineers, including Anthony of Naples, Hans Wolf, and Stefan von Haschenperg, but virtually nothing is known of these endeavors.^30^ Trade continued to be the main source of saltpeter in the first half of the sixteenth century. This changed during the reign of Elizabeth I when the English – distrustful of foreign merchants who sold saltpeter to their adversaries and wary of their mounting foreign debt and trade imbalance – heavily promoted domestic production by ramping up mining and the creation of artificial nitraries. Under the guidance of secretary of state William Cecil and financier Thomas Gresham, the crown sponsored all sorts of homegrown metallurgical, mineralogical, and alchemical projects from the 1560s onward.^31^ The Elizabethan government granted a slew of patents and monopolies to entrepreneurs in the 1570s and 1580s, and by around 1600, roughly half of all gunpowder expended by the English army contained saltpeter mined or manufactured in England.^32^

More important for early English entanglements with continental saltpeter producers were the numerous handbooks penned by mining and metallurgical experts and surreptitiously poached by English writers eager to abscond with this information across the Channel. These works recorded and codified existing practical knowledge, and they provide ample evidence of information exchange between chemical theorists and saltpetermen, alchemists and manual laborers, and natural philosophers and artisans. Writers of these metallurgical manuals like Vannoccio Biringuccio, Georg Agricola, Lazarus Ercker, and Peter Whitehorne tended to be dismissive of alchemy, or at least of the more grandiose claims of transmutational alchemists.^33^ This belies the sheer quantity of techniques, material knowledge, and equipment design borrowed from late medieval alchemical texts.^34^ For example, during the sixteenth century, small artisanal manuals (*Kunstbüchlein*) proliferated and circulated in Germany among both literate laborers who used them as practical resources and elite figures who often mined them for observational evidence of their published materials on alchemical theory.^35^ One of the first of this genre – the *Rechter Gebrauch d’Alchimei* (*The Proper Use of Alchemy*) of 1531 – explained that “true” alchemy was not about creating the philosopher’s stone to transmute base metals into gold, but rather the industrial methods of skilled workmen. These included many procedures used in the saltpeter industry, like the extraction of spirits from metals, the distillation of liquids to extract useful matter within, and the fixing of various substances, like mercury, sulfur, ammonia, and salts, that tended to be lost when materials were heated.^36^ Compiled from workshop notes, the oral transmission of artisanal processes, and earlier unpublished treatises, these small booklets, written in vernacular languages rather than Latin, provided instructions for operational techniques and standardized trades, and provided foundational access for apprentices.

Writers of later English alchemical manuscripts borrowed from these more conventional texts, and knowledge about the preparations of metals began to intersect with knowledge about the preparation of salts. In one anonymous text from ca. 1604, the author discussed saltpeter as a by product of the use of vermilion – a red pigment derived from cinnabar, used in printing and painting – in alchemical recipes for separating gold or silver from aqua fortis: a small amount of vermilion added to lye could reputedly be boiled until only saltpeter remained.^37^ After around 1600, short alchemical recipes to produce saltpeter and other rock salts, or longer recipes in which these were byproducts, clearly appropriated many of the best techniques from metalworkers, salt smiths, and other mining or military-oriented occupations. One undated manuscript from the early seventeenth century containing a recipe to create the vegetable stone, an alchemical substance alleged to stimulate growth, began with practical instructions explaining how salts were to be “drawn out of metals and to be turned into oyle and thereof to make the stone” in what appears to be a method for creating vitriols.^38^ Another manuscript containing alchemical recipes from around the same time covered how to distinguish salts from one another, their “chemical definitions,” and techniques for extracting them from plants.^39^ All these recipes and instructions date to after the later sixteenth-century development of a domestic saltpeter industry in England, and are based on practical techniques and matter theory, likely borrowed from both the late medieval alchemical tradition and the short metallurgical manuals of the sixteenth century.

**Figure 1.**
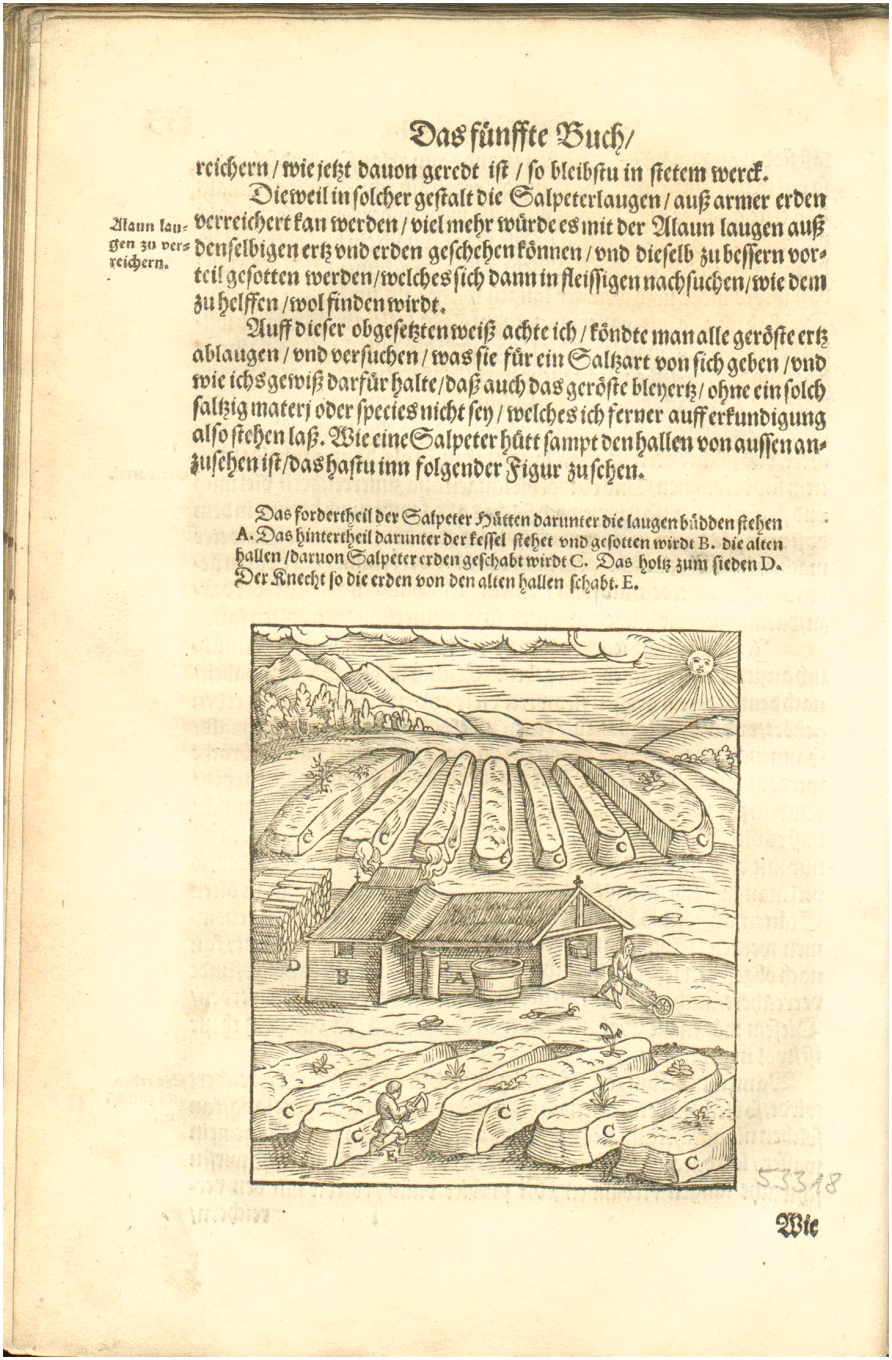
Niter beds with plants growing atop them. Lazarus Ercker, *Beschreibung allerfürnemisten mineralischen Ertzt und Berckwercksarten* (Frankfurt: Joannem Schmidt, 1580), fol. 133v. Photo: Deutsches Museum, München, Bibliothek, Sign.: 3000/1912B718.

Alchemy proved to be both the beneficiary and the driver of the early modern surge in saltpeter knowledge. As William Newman has shown, multiple factors coalesced in the fifteenth and sixteenth centuries to move alchemy from a peripheral position in the European scholarly landscape to a central one. While Renaissance humanists tended to deride alchemy, Neoplatonist philosophers sympathetic to natural magic like Marsilio Ficino, Giovanni Augurelli, and Heinrich Cornelius Agrippa asserted the existence of a universal life spirit (*spiritus mundus* or *spiritus vitalis*) extant in all matter and contended that alchemists might be able to experimentally isolate this.^40^ Those who worked with saltpeter were already fully aware that it nourished plant life in some way and intuited that something essential for the existence of life inhered in it. Woodcuts from Ercker’s *Beschreibung allerfürnemisten mineralischen Ertzt und Berckwercksarten* depicted artificial saltpeter beds thick with saplings and weeds alongside laborers with scythes to trim them lest these plants remove the fecund materials necessary to create saltpeter (Figure 1).^41^ Further changes to alchemical theory helped to integrate these separate bodies of knowledge.

Prior to the early sixteenth century, the “mercurial school” dominated Western alchemy. This dated back to the eighth-century works of the Arab alchemist Abū Mūsā Jābir ibn-Ḥayyan, known in the Latin West as Geber, and the thirteenth-century works of pseudo-Geber, which persisted well into the early modern era, when the Geberian mercury–sulfur theory of metallic composition became the cornerstone of European alchemy.^42^ In this tradition, all metals were believed to be composed to varying degrees of “philosophical” mercury and sulfur, immaterial substances that combined naturally underground to form the base metals of copper, iron, tin, lead, and quicksilver and the noble metals of silver and gold. The proportion of each determined which metal formed, and the experienced alchemist could alter these proportions in the laboratory to transmute one metal into another. For those whose alchemical objective was the transmutation of base metals into gold, mercury was the starting point for the creation of the philosopher’s stone, the object that made such transmutation possible.

But some alchemists argued for other starting materials. By the early sixteenth century, the idiosyncratic physician and alchemical theorist Paracelsus had added salt to the Geberian duo of mercury and sulfur to create what he called the *tria prima*, or three principles, the combination of which accounted for the composition not just of all metals but of all matter.^43^ Based in part on Paracelsian matter theory, many later sixteenth-century alchemists developed the *sal nitrum* theory, which posited that saltpeter, and not mercury, should be the starting material for the philosopher’s stone. Within a generation, the *sal nitrum* theory began to rival the mercurial theory, and these alchemists argued that unlocking the chemical secrets of saltpeter provided the key to understanding plant, animal, metal, and mineral generation and growth and the vital principles that made all life possible.^44^ In seventeenth-century England, agricultural reformers set about applying both the *sal nitrum* theory and the technical practices of both alchemists and saltpeter laborers to experimentally enhance agricultural productivity.

## The *sal nitrum* school in the Hartlib Circle: Sources, theory, and practice

Members of the Hartlib Circle hoping to convert saltpeter plants into artificial fertilizer production centers frequently deferred to *sal nitrum* theorists for their expertise. The most influential was Michael Sendivogius, whose 1604 work *Novum lumen chemicum* (first translated into English by John French in 1650 as *New Light of Alchymie*) provided the intellectual foundations for the *sal nitrum* theory among seventeenth-century alchemists.^45^ In what Thomas Leng has described as a “sexualized organic cosmology,” Sendivogius explained mineral growth, the nourishment of plant and animal life, and the procreant properties of soil through a cosmic process that produced the vital force found in saltpeter.^46^ Sendivogius argued that physical saltpeter was not the equivalent of the Neoplatonic *spiritus vitalis* but that it contained within it “seeds” – alternately referred to as *semina* and *sperma* – that were responsible for growth, broadly defined, in all matter.^47^ He described how these seeds descended to the center of the Earth, which was a hollow place much like a womb, where a second, inner sun (*sol centralis*) digested and vaporized them, after which they rose back through a semi-porous Earth to the surface.^48^ During their upward ascent they combined with philosophical sulfur in the soil, which accounted for both the formation of metals and minerals and the fertile properties of topsoil. Once airborne, these vaporous seeds traveled through the atmosphere, where the sun’s rays imparted their own vitalizing power, after which they condensed and returned to the Earth in various liquid forms, including rainwater, snow, and dew. At this point, after mingling with soil, the revitalized seeds produced ordinary saltpeter, the material substance in which these seminal forces that made life possible were concentrated.^49^ Sendivogius ended this explanation by noting that through this process “the greater quantity of salt-nitre [saltpeter] is made, and by consequence the greater plenty of corn grows, and is increased, and this is done daily.”^50^ According to Sendivogius, the same cosmic process that created saltpeter also made the growth of all plant life possible.

Another important *sal nitrum* theorist influential among the Hartlib Circle was the French alchemist and physician Joseph Duchesne (Latinized as Josephus Quercetanus).^51^ His immense popularity and notoriety led the English minister Thomas Tymme to translate large sections of his two most recent works, *Liber de priscorum philosophorum verae medicinae material* (1603) and *Ad veritatem hermeticae medicinae ex Hippocratis vertumque decretis ac therapeusi* (1604), to produce *The Practice of Chymicall and Hermeticall Physick* (1605).^52^ Duchesne drew inspiration from Paracelsian matter theory and Neoplatonic philosophy, which led him to argue that “the soule of the body and the world are knit together by meanes of the Aetherial Spirits going betweene, joyning each part to the whole into one substance.”^53^ These “aetherial spirits” were “vital forces” that pervaded the “astral seeds” (*semina*) found in all matter and provided evidence for the existence of philosophical mercury, sulfur, and salts in matter, as manifested most prominently in the physical substances bearing those names.^54^ Like Sendivogius, Duchesne raised salt to the highest prominence among the triumvirate and emphasized the importance of saltpeter for vegetation. Unlike Sendivogius, Duchesne argued that saltpeter itself had sulfurous, mercurial, and saline qualities, dependent upon how it behaved and where it could be found.^55^ As the *sal nitrum* school developed within the Hartlib Circle, adherents evoked the sulfurous aspects to explain saltpeter’s combustible properties, while they argued that its saline aspects – which Duchesne called the “vegetating soul” in plants – described its fertilizing properties.^56^ This notion that saltpeter animated matter differently in different substances, and that it exhibited sulfurous, mercurial, and saline characteristics that could be harnessed through alchemical experimentation for practical endeavors, is key to understanding how later members of the Hartlib Circle used this knowledge.

Many members of the Hartlib Circle accepted these theories as the explanation for soil fertility and plant generation and asserted that it could be controlled. For instance, Cheney Culpeper, a Kentish landowner and financial supporter of many agricultural experiments, kept a list of questions alchemists should keep in mind when attempting to purify metallic substances or recreate nature’s “Metalline and Mineral productions” in the laboratory.^57^ Referring to the works of Sendivogius, Culpeper asked whether “there is a real vegetative Life in Mettals equal to that in other Bodies. . .wherein this life principally consists. . .how to actuate this life. . .how to make it a more living life,” and whether this pertained to a vital activator “that ought bee in Metals and Minerals as in other Bodies and Seeds.”^58^ He went on to agree with Sendivogius’s assessment that atmospheric waters contained within them seeds that had been revitalized by the sun and that these accounted for the growth of both mineral and vegetable substances.^59^

In letters to the physician Benjamin Worsley, Culpeper extolled the writings of Sendivogius and other alchemists and natural philosophers who had argued in favor of vitalist interpretations of matter. Culpeper posed to Worsley a series of questions, hoping to answer whether the cosmic process that created the vitalizing power of saltpeter might be replicated in the laboratory or on the farm, in order to control the “exilation of *th*e Spirit of Nature againe and againe.”^60^ He related the opinions of several natural philosophers, one of whom was the unnamed author of “*Traitte de Sel*,” presumably meaning *Traittez de l’Harmonie et Consitution generalle du vray sel* (*Treatise of the Harmony and General Constitution of the True Salt*), a plagiarized work published by the Sendivogian alchemist Clovis Hesteau, Sieur de Nuysement but derived entirely from Jean Brouaut’s *Trois livres des éléments chymiques et spagyriques*.^61^ In this letter, Culpeper essentially suggested that the atmosphere was like a giant, natural alembic that had distilled a vaporous “aerial niter,” causing the seeds to return and “heat” or animate water, beginning the process that explained vegetative growth.

Hartlib’s group held Sendivogius in extremely high regard. In an anonymous pamphlet in Hartlib’s possession on the “question concerning fertility,” the author placed Sendivogius alongside Aristotle, as well as contemporary alchemists and natural philosophers like Jan Baptist Van Helmont, René Descartes, Kenelm Digby, and Johann Rudolf Glauber, as the foremost authorities on the question of whether “spermatic vapors [rose] from the center of the earth. . .or [came] from heavenly influence,” whether “water was impregnated” by some vital seed, or whether the earth had “infinite strength” or the “infinite power of the prolific, fiery *semina*, whose wealth shall never be exhausted.”^62^ According to Culpeper, Worsley, and Hartlib, the key to developing an inexhaustible material wealth of fertile soil through the production of saltpeter depended first on learning how nature produced saltpeter and then upon harnessing that knowledge to create fertile soil.

Benjamin Worsley rapidly became the resident saltpeter expert among the Hartlib Circle.^63^ His library contained three works by Paracelsus, several alchemical texts by Peter Severinus, Joseph Duchesne, Jean Beguin, Angelo Sala, and Johann Hartprecht, as well as De Nuysement’s *Traittez. . .du vray sel* and the *Traicté du Feu et du Sel* (*Tract on Fire and Salt*) by Blaise de Vigenère, both explicitly Sendivogian works that had been translated into English in the mid seventeenth century.^64^ Worsley saw in Sendivogius and his followers a way to recreate the natural processes of chemical transformation artificially. In *Novum lumen chemicum*, Sendivogius had suggested that seminal qualities existed not just in physical materials that could “grow,” like metals and plants, but in “the Elixir of everything or quintessence or the most perfect decoction or digestion,” which nature produced and which the adept alchemist could learn to replicate.^65^ In fact, Worsley seemed to think that this was the actual, ultimate task of artificial saltpeter manufacturing. “For as mutch as in all mineralls and metals there is a participation of the same lyfe,” Worsley wrote in a letter, probably to Hartlib’s alchemist son-in-law Frederick Clodius,

blessing vegitatiue and multiplicatiue virtue, as was given in the creation of two plants and other seed-bearing boddyes by reason the said vegitatiue virtue or spirritt is to *th*e outward sense imprisoned and not to bee diserned vntill brought forth in or by this Mercuriall substance hence the same substance before cald water is cal’d a Sperma or *Anima cuiuslibet Mettali* [a soul which pertains to a metal].^66^

In short, Worsley believed, following Sendivogius, that the same vital force that caused metallic generation also accounted for botanical generation via a philosophical niter that materially manifested in topsoil as saltpeter. Where he went one step further was to suggest that this process could potentially be replicated and controlled. Attempting this process with saltpeter, first in the laboratory and then in the field, animated his professional life for much of the 1640s and 1650s.

Worsley became connected to the Hartlib Circle in May 1644. Very quickly, he began corresponding voluminously with alchemists in Hartlib’s orbit including Robert Boyle, Frederick Clodius, Johann Moriaen, and Gabriel Plattes, among others, on all things related to the production of saltpeter.^67^ In particular, these figures had become highly interested in developing a method of creating saltpeter without the unpalatable work of digging for nitrous earth in latrines, poultry coops, and barnyards or working directly with animal dung and urine on-site at nitraries. By the summer of 1644, Worsley was collaborating with Hartlib and other members of his circle on a project to supply Parliamentary forces – then engaged with the Royalist army during the English Civil War – with saltpeter to make gunpowder.

In service of these goals, Worsley embarked upon a venture to develop the requisite technical skills. Following several false starts in the mid-1640s, he secured funds to travel to Amsterdam in late 1647 to study alchemy under the illustrious adept Johann Rudolf Glauber, an expert in saline chemistry who had set up an alchemical furnace and laboratory there three years earlier.^68^ Worsley learned from Glauber about both the chemical and medicinal properties of salts and worked as an assistant in his laboratory, studying alchemical techniques like calcination and distillation. With distillation, one of the quintessential alchemical laboratory processes, Worsley acquired the knowledge to condense “sprits,” usually in liquid form, but which early modern alchemists, including Glauber, had learned how to crystallize into salts.^69^ This same technique was indispensable to saltpeter boilers working at nitraries. Ultimately, however, Worsley’s time in Amsterdam was short-lived. After repeated failure to discover a universal solvent – and his own disillusionment with Glauber’s self-proclaimed successes – he returned to England after fewer than two years with the hope of securing a state position.^70^

After Worsley returned to England, Hartlib encouraged him to continue his research into the “vtopian designs. . .for [an] Artificiall way of breeding [&] increasing of Salt-Peter” without having to resort to the use of “Dung, Urine, and the like stuff.”^71^ Despite his misgivings about Glauber, Worsley claimed to be able to produce saltpeter alchemically without these substances after working with him. Perhaps most importantly, Worsley believed that his research into saltpeter itself had led to knowledge not just of its chemical nature but “something also of. . .[all] vegetation.”^72^ Worsley’s field experiments on saltpeter led him to the conclusion in *De Nitro Theses Quædem*, his unpublished treatise on the topic, that “all Plants containe in them Salts” and that “nature’s intent in the breeding of Salt-Peter in the Vpper Surface of the Earth is for the generation of Plants and by them for the præservation of Animals.”^73^ This prefigured Glauber’s own assertion two years later that “Salt-Peter was a necessity in the Herbs, & the Grass, afore the Beasts feeding on them.”^74^ Worsley’s final experimental results in the field seemed to confirm this:

It is found by certain and frequent experience that Salt-Peter is the very soule of Vegetation as may appeare by Corne or Seedes steeped in Water mixed with Salt-Peter, w*hi*ch by several trials is found to bee the best imbibition of any. As also by all the Earth rich in Salt-Peter, w*hi*ch is found fatter and richer in Spirit, then any other Compost in the World made by what Art soeuer, w*hi*ch is further confirmed by the ma*n*ner of Vegetation or Germination, w*hi*ch consists in the dissolution and apposition of Salt or any Salinous matter. . . It is found by the certainty of Experience that all such Earth as is once imprægnated or enriched w*i*th a nitrous Spirit, ceaseth not to generate and multiply its*elf* vpon all such fit matter as shall bee apposed to it, prouided shee bee not hindred.^75^

That is, regardless of whether or not one could equate it with a universal vital spirit, since it appeared wherever plant life could be found growing, saltpeter was the key to understanding all botanical growth and, through ingestion, human and animal nourishment as well.

## “Rusticall chymistry” and saltpeter experiments: Hartlib Circle agricultural projects with artificial fertilizers

Research and experiments like those undertaken by Worsley became increasingly common among Hartlib Circle agricultural reformers in the 1650s. Earlier experimental trials with saltpeter – notably those performed in the early seventeenth century by Hugh Plat and Francis Bacon – tended to be small-scale investigations of its effects on individual seeds.^76^ Those in Hartlib’s network planned much larger projects to create new nitraries, repurpose older ones, and use the saltpeter generated to fertilize farms on a massive scale. Although none of these plans came to fruition, this decade was a heyday for experimental agriculture rooted in saline chemistry. Specific historical circumstances coincided to allow this: as noted in the previous section, the vitalist, *sal nitrum* alchemical theory offered a powerful alternative to the mercurial school, and most of Hartlib’s associates involved in alchemy subscribed to some version of this theory. By September 1651, the English Civil War, which had riven the isles for nearly a decade, came to end. While the threat of resumed violence – not to mention war with the Dutch – loomed throughout the 1650s, the immediate need for copious amounts of saltpeter for gunpowder subsided. Chronic shortages remained an ever-present fear (and replenishing exhausted stores in peacetime was standard procedure), but by mid-century the English East India Company (EIC) rapidly became the primary supplier of saltpeter to the country.

Though saltpeter had become an abundant commodity on the international market by the middle of the seventeenth century, it had also come almost entirely under the purview of state actors and their allies. In England, the EIC overtook foreign imports, domestic mining, and domestic manufacturing plants in the late 1630s, just as Hartlib and his associates began investigating saltpeter’s fertile properties in earnest.^77^ It first turned a profit in 1643 and controlled (or coerced) enough Indian refineries to manufacture high-quality saltpeter in perpetuity around 1644.^78^ Beginning in the 1620s, EIC ships, imitating their Dutch rivals, began using saltpeter instead of rocks for ballast. Its attributes as a preservative and insect repellant helped to extend the life of ship hulls and protected perishable cargo like tea, indigo, and various spices. As early as 1639, the company had to regulate the amount of saltpeter they shipped to England for fear of flooding the market and crashing the price.^79^ These economic developments suggest that the influx of foreign saltpeter, managed and consumed by the military, first alleviated the pressure on the domestic industry to produce enough quantities for gunpowder and then obviated its need altogether.^80^ This freed up entrepreneurs, projectors, and craftsmen to become increasingly experimental and reappropriate saltpeter for other uses. Given its known capacities as a fertilizer, agriculture became a natural outlet.

Because artificial nitraries created saltpeter from abundant, inexpensive materials like animal manure, urine, dead plant matter, and nitrous soil from farms and outhouses, one obvious question arises: why not simply use these materials as fertilizers in the first place? The answer to this was twofold. First, many agricultural reformers connected with Hartlib were also social and economic reformers who believed that human ingenuity and the utilitarian sciences championed by figures like Francis Bacon could create agricultural bounty irrespective of any supposed natural limits. Thus, they believed that experimentally manipulating these base materials using the latest scientific knowledge could potentially enhance the fertility of these substances exponentially.^81^ Secondly, alchemy promised precisely such power, and the notion of an unlimited supply of saltpeter was consistent with Hartlibian interpretations of fertilization as a form of alchemical multiplication.^82^ This is what Worsley meant when he claimed he could replicate the natural growth of saltpeter in an artificially controlled environment to coax out the “nitrous Vniversal spirit,” which Robert Boyle later lauded as the possibility of a “perpetual mine of salt-Petre.”^83^ In his *Sceptical Chymist*, Boyle promoted the theory that “the seminal principle of nitre, latent in the earth, does, by degrees, transform the neighbouring matter into a nitrous body,” meaning that small amounts of saltpeter could “seed” mixtures and be alchemically multiplied to continuously replenish soil.^84^ Borrowing from the alchemical concept of multiplication (*multiplicatio*) – one of the keys to creating the philosopher’s stone – agricultural reformers argued that small amounts of saltpeter placed under the proper chemical conditions would “reproduce” on their own.^85^ Both Boyle and Worsley advocated for this process.

Throughout the 1650s, in large part due to Worsley’s earlier work, Hartlib and many of his correspondents expanded their support for projects and experiments designed to create saltpeter artificially or augment extant saltpeter in just this way. In marginal notes from a January 1650 journal entry on “secrets to growing saltpeter,” Hartlib summarized meeting with a clergyman who claimed to have knowledge of saltpeter production under the subheadings “Salt-peter,” “an Experiment for Pasturage to make Agriculture Husbandry,” and “the Dunging of Land.”^86^ In these notes, Hartlib wrote that Paracelsian physician Johann Brün made “grasse growe thrice within three months” in “an Experiment for the dunging of Land without dunging.”^87^ Presumably, Brün was using a type of base matter other than manure, urine, or vegetable matter, just as Worsley had claimed was possible after his time with Glauber in Amsterdam.

Some farmers attested to this possibility. Peter Smith wrote to his uncle, apple orchard proprietor and alchemical experimenter John Beale, that if one followed certain husbanding procedures, saltpeter would actually be a natural by product, thus creating a potentially endless cycle of fertile ground in the same location. According to Smith, it was as simple as dunging the land with manure as usual but, after harvesting the crop, returning to the field again to collect the saltpeter that came from the remaining manure. He wrote that “dryness,” meaning soil with little nitrogenous materials, “conduceth much to *th*e increase of Niter (w*hi*ch [Francis] Bacon calls *th*e Spiritt of earth) as may be demonstrated by *th*e Saltpeter works.”^88^ Soil without much saltpeter, Smith argued, could be seeded with manure to create it. To make this process productive, Smith suggested that “the best meanes I have found to spare compost” was by “burning the halfe of my tillage, w*hi*ch being thereby sufficiently manured, doth surrender its share of dung to *th*e other halfe, whereby I have some plenty of compost for orchards, garden & pasture.”^89^ Much like Boyle’s “perpetual mine of saltpeter,” this method promised a potentially limitless supply of fertile, arable ground, provided there was a steady stream of animal manure and proper management of the land at harvest time.

Hardly a month went by in the early 1650s in which Hartlib did not receive news of some new process for creating saltpeter artificially or a better method of a known process. Even as various alchemical schemes to create saltpeter failed, Hartlib continued to have faith that a chemical manipulation of the essential ingredients of saltpeter provided the best path forward to a universal, artificial fertilizer. In journal entries from March through May of 1653, Hartlib noted that a former Fellow of Trinity College, Cambridge, named Mr. Jolly, whom he described as “a kind of Rosæcrucian or Adeptus,” had been working with a well-known saltpeterman named Mr. Worthington at Salisbury Court to create saltpeter artificially simply by adding small amounts of it to potash and allowing to it multiply naturally. After an unnamed amount of time, “a certain quantity of salpeeter put amongst it. . .turns all the rest of the pot-ashes into salpeeter.”^90^ This process was similar to one described by Johann Moriaen, who had been the go-between for Worsley and Glauber in Amsterdam.^91^ Just as Smith, Jolly, Worthington, and Boyle had contended that saltpeter would multiply given the proper medium in which to grow or with the addition of a single, simple ingredient, Moriaen claimed to have made his own observation about a certain “ferment” that, when added to an appropriate “fit matter. . .will cause the whole at lengtht to turne into the Nature of nitrium” in a manner that mirrored its natural growth.^92^ This inexpensive and abundant “fit matter” was composed of grass clippings and seeds, sometimes mixed with lime or wood ash, while the ferment was the richest earth one could find, which was “impregnated with its own nitrous Spirit.”^93^ Moriaen’s technique had less regard for environmental conditions because, he wrote, “no season or weather shall hinder its fermentation.”^94^ By fermentation, Moriaen referred to the process described by a number of vitalist alchemists including Paracelsus, Van Helmont, and Duchesne, meaning the transformation of a material mass into a qualitatively more perfect and quantitatively more numerous substance.^95^ Through this method, Moriaen promised “in a short time. . .a thousand hundred” yield from “one of good and excellent Salt-Peter.”^96^

The later 1650s witnessed a decline in fervor over these saltpeter projects. While agricultural reformers continued to exalt its potential as a universal fertilizer, the failure to establish an infrastructure to create and distribute saltpeter for these purposes soured many reformers on the possibility of radically transforming English agriculture. Worsley ended his attempts to alchemically manufacture saltpeter and gave up experimental natural philosophy altogether in the mid-1650s.^97^ Robert Boyle began to collaborate with the American alchemist George Starkey, who scorned the *sal nitrum* school in favor of the older mercurial tradition.^98^ Meanwhile, Hartlib turned his attention to creating saltpeter from seawater in conjunction with contemporary desalination projects, and he wrote excitedly to Boyle, the Dutch historian and alchemist Georg Horn, and the natural philosopher Henry Oldenberg about them, but little seems to have come of these.^99^ Hartlib died in 1662. Experimental analyses of plants, seeds, soil, and saltpeter continued among natural philosophers with the establishment of the Royal Society in 1660 and the creation of the Georgical Committee on agricultural affairs in 1664, yet there is little evidence that this research led to any major attempts to improve agriculture with alchemy.^100^

## Legacies of agricultural experiments with saltpeter: Husbandry manuals in the later seventeenth century and beyond

Ultimately, the major projects that members of the Hartlib Circle attempted to implement were disappointments. They constructed no new major saltworks in the 1640s and 1650s and, although Worsley succeeded in purchasing workable recipes for its production, there is no evidence that any of them developed into anything more than small-scale schemes.^101^ Yet, the experimental culture that pervaded the Hartlib Circle’s efforts paralleled farmers’ hands-on uses of saltpeter first as a fertilizer and later as a chemical irritant that drove away insects, vermin, and pests from seeds and provided resistance to molds, fungi, and smut diseases.^102^ This legacy persisted in the many husbandry manuals published across the English-speaking world in large numbers from the mid seventeenth century onward, many of which were authored by Hartlib’s colleagues. Although instructional farming tracts dated back to the ancient Greco-Roman world and the English genre originated in the early sixteenth century, they proliferated at a far greater rate after around 1650.^103^ There is little evidence that common farmers regularly consulted these didactic texts. However, husbandry manuals and horticultural texts from around 1650 until the end of the eighteenth century often cited saltpeter as an agriculturally important product, which suggests that farmers’ applications of it bore some resemblance to mid-seventeenth-century Hartlibian experiments.

Already by 1600, English agricultural sources had noted the importance of saltpeter for plant growth. Hugh Plat had argued that manure was such a potent fertilizer because it contained within it a “vegetable and fructifying salt of Nature” responsible for growth, which he asserted could be replicated artificially.^104^ Elsewhere he wrote that steeping seeds or sprinkling them with mixtures of dung and saltpeter could increase the yields of cereal grains, and he developed an elaborate alchemical explanation for this.^105^ Francis Bacon also experimented with saltpeter as a seed steep and fertilizer in his *Sylva sylvarum*. Though he hesitated to equate saltpeter with the vital power of the most enthusiastic *sal nitrum* theorists, Bacon did single out saltpeter for its usefulness in accelerating germination, writing that seeds sprouted due to the “spirit of the Nitre; for the Nitre [is] the Life of Vegetables.”^106^ Both alchemical interpretation and agricultural applicability characterized these early texts of experimental husbandry.

Hartlib and his associates took these examples to heart. In a memorandum from February 6, 1652, Hartlib noted that it would be “useful to the human race if we could multiply corn [grain] artificially,” and he suggested, based on the work of various correspondents, that a steeping liquid to soak seeds could be “invigorated with a Lixivium,” a solution containing alkaline salts like saltpeter extracted from wood ash or leached from charred timber, usually using lye.^107^ Around the same time, in his pamphlet titled “A Discovery for Division or Setting Out of Land,” agricultural reformer Cressy Dymock relayed information about a French experiment “for the multiplying of corn,” in which saltpeter was dissolved into a liquid solution of rain water, cow dung, pig dung, and pigeon muck. Steeping seeds in this mixture and planting in barren ground “produced unusuall increase” where, Dymock claimed, “one hundred and fourteen eares upon one root. . .came from one single corn so prepared.”^108^

Experimental trials like these featured prominently in his *Legacy of Husbandry*, an edited compilation of writings on agricultural improvement, to which his associates, including Gabriel Plattes and Arnold Boate, contributed. Attempts at fertilizing farm fields prompted Plattes, for example, to devise an experiment “wherein is showed how a rich compost may be made in the form of Earth. . .which may be converted into saltpeter.”^109^ He cited a saltpeterman’s report of saltpeter dissolved in water as the source of “the best Liquors. . .to be got” for fertilization. Seed steeps and “fructifying waters” – large vessels containing seeds soaking in water, dungs, and chemical solutions – evolved out of these joint alchemical and agricultural ventures. Saltpeter featured prominently in many of them.

One of the most vehement promotions of saltpeter as the most essential ingredient in seed steeps came in John Worlidge’s *Systema Agriculturæ* of 1668. In this work, Worlidge touted the uses of “steeping *Corn* in Dung-water,” as supported through the experience of many farmers; yet he warned that the agricultural reformer with alchemical knowledge was likely to have much grander expectations than the country farmer and might be disappointed with the ultimate yield. Instead, Worlidge promised that his way, reinforced by techniques from contemporary chemistry, was more “excellent,” “grounded on more rational Principles,” and “more effectual” than any other. He wrote, “that which containeth in it most of the *Universal Subject* or *Matter* of *Vegetables*. . .is the fittest for this purpose; of all which, *Nitre* or *Sal terrae* is esteemed the best, wherewith *Virgil* adviseth to infuse or besprinkle the seed.”^110^ Worlidge cited the experimental work of Glauber to bolster his assertions and claimed that this “menstruum” both ripened grains quicker and improved the chances of each individual grain bearing a healthy plant. The same mixture supposedly worked when applied directly to the roots or through simple irrigation to a young plant. However, according to Worlidge, the niter that was best suited for this task was not natural saltpeter efflorescing in caves or mined from dry salt flats. Again, quoting Glauber, Worlidge alleged that:

Common Nitre [was] not fit for that purpose. The *Nitre* or *Sal terrae* intended by these and other Learned Authors as apt for this work, is the fixed Salt extracted out of any *Vegetable, Animal*, or *Mineral* throughly calcined, as after the burning of Land in the common way of *burn-baiting*, that which causeth so great Fertility is as well the fixed *Salt* or *Alcali* that’s left in the *Ashes*, as the waste or expence of the sterile acid Spirit which before kept that vegetating Salt from acting. What is it that is fertile in Lime, Ashes, Soap-ashes, *&c.* but this *Nitre*, or *Sal terrae*, this *Universal Subject* left therein, and most easily separable after calcination?^111^

Calcination had long been a well-known alchemical procedure in which a mineral or metallic substance was heated over a very hot flame until much of the original material had burned away.^112^ However, calcination had become a common practice in experimental plant chemistry in the sixteenth and seventeenth centuries as well, especially among *sal nitrum* theorists, since various salts and alkaline materials often remained in the residue of charred vegetable matter. Here, Worlidge, like many from the previous generation of *sal nitrum* theorists, simply noted that the salts found in plant or animal matter demonstrated that they had already been essential to sustain life and thus reintegrating them into the soil or water used to steep and nourish newly planted seeds was akin to manuring a field with animal dung. By extracting it from plant matter and infusing it directly back into the seed or the soil, farmers simply skipped a step and accelerated this natural process.

Brining, liming, and steeping seeds in chemical mixtures made up primarily of water and various salts continued throughout the late seventeenth century and into the eighteenth century. However, in contrast to earlier seed steeps, later recipes almost universally praised saltpeter not for its fertilizing potential but for its pesticidal properties. In the 1650s, agricultural improvers Walter Blith and Adolphus Speed had both recommended steeping wheat with common saltwater to prevent smut and bunt, fungal infections common to cereal grains.^113^ Toward the end of the seventeenth century, agricultural writers suggested similar uses for saltpeter, which morphed from a fertilizer to a pesticide, first against fungal blights and then against soft-bodied invertebrates like worms, slugs, and snails. In his 1675 *Planters Manual*, Charles Cotton mentioned it specifically for this purpose, particularly on branches and leaves.^114^

In 1721, John Mortimer advocated mixing saltpeter with sheep’s dung, alum, and urine as a preventative for disease.^115^ The celebrated early eighteenth-century agriculturist Jethro Tull simply argued that brining seeds after treating them with quicklime had a similar effect on seeds as it did on meat and could be a potent preservative to guard against premature decay.^116^ In 1756, Thomas Hale argued for the addition of copperas to saltpeter brine to defend roots against burrowing worm attacks.^117^ Matthew Peters’s recipe for seed preservation from 1771 contained saltpeter along with lime, alum, verdigris, vitriol, plant ash, and common salt.^118^ These practices seem to have died out between the late eighteenth and early nineteenth centuries as a result of French research into the fungal maladies of wheat seeds, which showed lime to be instrumental in warding off infection and brines of saltpeter or common salt to be of little or no use.^119^ At a purely practical level, even stripped of the alchemical or agricultural reason for steeping, soaking seeds in saltwater caused sterile seeds, weed seeds, and seeds hollowed out by worms and insects to float, and farmers could skim them from the surface and discard them, ensuring minimal waste of valuable field space.^120^ Saltpeter’s return as a fertilizer would have to wait until scientists isolated potassium and nitrogen as macronutrients in industrial chemical agriculture in the late nineteenth and early twentieth centuries.

## Conclusions and further investigations

Manufacturing saltpeter for gunpowder in the early modern world was, as Brenda Buchanan put it, both an “art and a mystery.”^121^ It was also a science. This science of saltpeter obtained knowledge from many sources, including miners and metallurgists, military engineers, nitrary laborers, agriculturists, botanists, and practicing alchemists. Beyond its integral role in gunpowder, saltpeter possessed multifarious properties that allowed for a wide range of uses in everything from medicine to food preservation to trades like dyeing, glassmaking, and engraving. Its importance for experimental agricultural improvement is one of its overlooked functions, particularly in seventeenth-century England.

Though observers in the early sixteenth century were clearly aware that saltpeter was connected in some way to soil fertility and plant growth, only in the seventeenth century did natural philosophers, alchemists, and agricultural reformers initiate an experimental regime designed explicitly to determine the source and nature of that vitalizing power. This would not have been possible without several contingent factors: the rise of artificial saltpeter manufacturing plants to supplement natural reserves; the expansion of vitalism into alchemical practice and matter theory; and the later dominance of the English East India Company in the saltpeter trade. Together, these created conditions allowing the Hartlib Circle to attempt a repurposing of saltpeter as an artificial fertilizer. Though these attempts failed to deliver anything transformative on a grand scale, the knowledge garnered from these trials proved important for agricultural improvement from the second half of the seventeenth century onward.

Much work remains to be done before historians gain a fuller picture of the intersections between the saltpeter industry, alchemy, and agriculture in the early modern era. In the mid-seventeenth-century English context, we find Hartlib’s group hoping to unite the labor and knowledge of the domestic saltpeter industry with agriculture. This relationship was seldom rosy. Across Europe, governments authorized saltpeter diggers to remove saltpeter from farms, much to the chagrin of landowners. Cadastral surveys, tax records, and state documents detailing the legal rights of saltpetermen demonstrate the exploitative and rapacious nature of those who, on behalf of the state, obtained nutrient-rich raw materials for the Ordnance Office that would otherwise have remained in the ground for farmers. By law, saltpetermen were instructed to reimburse landowners for any property damage or loss of income, but in practice this seemed rarely to have been the case, and there is evidence of farmers negotiating with, bribing, and even violently confronting saltpetermen when they made their rounds.^122^ Further work on the social and legal relationships between laborers in the saltpeter industry and farmers is necessary to evaluate just how feasible the Hartlibians’ desire to reconcile these disparate interests were.

This competition was not limited to farmers and saltpetermen. Elsewhere, it manifested as a competition over available nitrates and resulted in a documented loss in soil fertility. Historians studying saltpeter production in early modern Austria–Hungary, France, and Sweden, for example, have noted the trade-offs between gunpowder and food security when allocating these resources.^123^ Sixteenth- and early seventeenth-century England appears to have faced the same painful ecological roadblock, yet, through the EIC, they eventually achieved saltpeter independence that not only provided state security but also reserved nitrogenous soil solely for farmers. In 1600, England domestically produced half of its saltpeter for gunpowder; by the late 1660s, it was less than five percent.^124^ Agricultural historians investigating the increase in crop yields and the decrease in food dearth during the early decades of the British Agricultural Revolution of the seventeenth and eighteenth centuries should note these comparative developments. Environmental historians conducting long-term socio-ecological analyses on agricultural ecosystems and their effects on the surrounding environment should also keep these developments in mind.^125^

Over the last two decades, historians of science have begun to investigate alchemy not only as a secretive, scholarly art performed by adepts in the laboratory but also as an applied, technical practice with implications for trade, commerce, and the formation of incipient pharmacological, chemical, and metallurgical industries. Members of the Hartlib Circle clearly engaged in what Tara Nummedal has described as “entrepreneurial” and “vernacular” alchemy in that they hoped to use cutting-edge alchemical knowledge to create a specific product for use in agriculture and inhabited social and cultural spaces typically populated by artisans, craftsmen, and tradesmen.^126^ In the post-Baconian landscape of mid-seventeenth-century experimental natural philosophy, open knowledge directed toward public good became an increasingly important value.^127^ The agricultural uses of alchemy provide further evidence of this. Abetted by the belief that human ingenuity could transcend natural limitations through the practical application of the sciences, Hartlibian agricultural alchemists sought to multiply saltpeter artificially in ways not envisioned prior to this era. This study adds to those histories of science devoted to exploring alchemy as an everyday makers’ knowledge practiced outside of the laboratory.

## Footnotes

1. Cheney Culpeper to Samuel Hartlib, February 18, 1653, 39/1/5A, Hartlib Papers (hereafter HP), University of Sheffield Library, <www.dhi.ac.uk/hartlib/> (August 1, 2018). Italics in the original. These are originally abbreviations within words and represent editorial choices made by archivists to improve clarity and readability. I have preserved these edits and original spelling throughout.

2. Peter Whitehorne, *Certaine Wayes for the Ordering of Souldiours in Battelray. . . and Moreouer, How to Make Saltpeter, Gounpouder and Diuers Sortes of Fireworkes or Wilde Fire. . .* (London: John Kingston for Niclas Inglande, 1562), p.22.

3. William Shakespeare, *Henry VI, Part 1*, David Bevington (ed.), (Oxford: Oxford University Press, 1987), 1.3.58-60, p.147.

4. William Clarke, *The Natural History of Nitre: Or, a Philosophical Discourse of the Nature, Generation, Place, and Extraction of Nitre, with its Vertues and Uses* (London: E. Okes for Nathaniel Brook, 1670), pp.19, 53.

5. David Cressy, *Saltpeter: The Mother of Gunpowder* (Oxford: Oxford University Press, 2013), pp.29–32; Haleigh Robertson, “Reworking Seventeenth-Century Saltpetre,” *Ambix* 63, no. 2 (2016): 145–61, 147–8.

6. Cressy, *Saltpeter*, esp. pp.9–35 (note 5). See also David Cressy, “Saltpetre, State Security and Vexation in Early Modern England,” *Past and Present* 212 (2011): 72–111.

7. James W. Frey, “The Indian Saltpeter Trade, the Military Revolution, and the Rise of Britain as a Global Power,” *The Historian* 71, no. 3 (2009): 507–54.

8. Robertson, “Reworking Seventeenth-Century Saltpetre” (note 5); Haileigh Robertson, “‘Imitable Thunder’: The Role of Gunpowder in Seventeenth-Century Experimental Science” (PhD diss., University of York, 2015); the Medieval Gunpowder Research Group, esp. “Making Saltpetre: Report 11,” 2013, <https://ahc.leeds.ac.uk/downloads/download/35/ho_medieval_gunpowder_research_group> (March 1, 2021).

9. Kelly DeVries, “Sites of Military Science and Technology,” in Lorraine Daston and Katherine Park (eds.), *The Cambridge History of Science*, Vol. 3, *Early Modern Science* (Cambridge: University of Cambridge, 2006), pp.306–19; Bert S. Hall, *Weapons and Warfare in Renaissance Europe: Gunpowder, Technology, and Tactics* (Baltimore, MD, and London: Johns Hopkins University Press, 1997), pp.xxiii–xxv, 42–3; Brett D. Steele and Tamera Dorland (eds.), *The Heirs of Archimedes: Science and the Art of War through the Age of Enlightenment* (Cambridge, MA: MIT Press, 2005); Brenda J. Buchanan (ed.), *Gunpowder, Explosives, and the State: A Technological History* (Farnham, UK: Ashgate, 2006).

10. There are some notable exceptions. See, e.g., Allen E. Smith and Diane M. Secoy, “A Compendium of Inorganic Substances Used in European Pest Control before 1850,” *Agricultural and Food Chemistry* 24, no. 6 (1976): 1180–91; Allen E. Smith and Diane M. Secoy, “Early Chemical Control of Weeds in Europe,” *Weed Science* 24, no. 6 (1976): 594–7; Allen E. Smith and Diane M. Secoy, “Salt as a Pesticide, Manure, and Seed Steep,” *Agricultural History* 50, no. 3 (1976): 506–16; Dino Güldner, Fridolin Krausmann, and Verena Winiwarter, “From Farm to Gun and No Way Back: Habsburg Gunpowder Production in the Eighteenth Century and Its Impact on Agriculture and Soil Fertility,” *Regional Environmental Change* 16, no. 1 (2016): 151–62; Antonio Clericuzio, “Plant and Soil Chemistry in Early Modern England: Worsley, Boyle, and Coxe,” *Early Science and Medicine* 23, nos 5–6 (2018): 550–83.

11. Cressy, *Saltpeter*, pp.130–5 (note 5); Frey, “The Indian Saltpeter Trade,” 507–11 (note 7); K. N. Chaudhuri, *The English East India Company: The History of a Joint Stock Company, 1600–1640* (London: Frank Cass, 1965), pp.189–90; K. N. Chaudhuri, *The Trading World of Asia and the English East India Company, 1660–1760* (Cambridge: Cambridge University Press, 1978), pp.336–41.

12. Ku-Ming Chang, “Alchemy as a Study of Life and Matter: Reconsidering the Place of Vitalism in Early Modern Chymistry,” *Isis* 102, no. 2 (2011): 322–9.

13. William Newman, “From Alchemy to ‘Chymistry,’” in Lorraine Daston and Katherine Park (eds.), *The Cambridge History of Science*, Vol. 3, *Early Modern Science* (Cambridge: University of Cambridge, 2006), pp.497–517.

14. See, e.g., Anna Marie Roos, *The Salt of the Earth: Natural Philosophy, Chemistry, and Medicine in England, 1650–1750* (Boston: Brill, 2007), pp.10–46; Robert R. Frank, Jr., *Harvey and the Oxford Physiologists: Scientific Ideas and Social Interaction* (Berkeley: University of California Press, 1980), esp. pp.142–94; Clericuzio, “Plant and Soil Chemistry in Early Modern England,” 551–4 and 564–8 (note 10).

15. Charles Webster, *The Great Instauration: Science, Medicine, and Reform, 1626–1660* (London: Duckworth, 1975), esp. pp.384–402.

16. James Delbourgo and Nicholas Dew (eds.), *Science and Empire in the Atlantic World* (London: Routledge, 2007), pp.1–4.

17. Charles Webster, *Samuel Hartlib and the Advancement of Learning* (London: Cambridge University Press, 1970); Mark Greengrass, Michael Leslie, and Timothy Raylor (eds.), *Samuel Hartlib and Universal Reformation: Studies in Intellectual Communication* (London: Cambridge University Press, 1994).

18. Vera Keller and Ted McCormick, “Towards a History of Projects,” *Early Science and Medicine* 21, no. 5 (2016): 423–44. On projects in early modern England more generally, see Koji Yamamoto, *Taming Capitalism before Its Triumph: Public Service, Distrust, and ‘Projecting’ in Early Modern England* (Oxford: Oxford University Press, 2018).

19. Peter Smith to John Beale, April 11, 1650, HP 67/23/2A–2B.

20. Joseph Needham, *Science and Civilisation in China*, Vol. 5 (Cambridge: Cambridge University Press, 1954), pp.179–200. See also Kenneth Chase, *Firearms: A Global History to 1700* (Cambridge: Cambridge University Press, 2003), pp.31, 58.

21. Michael Scot, *De alchemia* (*On Alchemy*) of ca. 1230; Roger Bacon, *Epistola de secretis operibus artiis et naturae, et de nullitate de magiae* (*Letter on the Secret Workings of Art and Nature, and on the Vanity of Magic*) of ca. 1248–67; [pseudo-?] Albertus Magnus, *De mirabilibus mundi* (*Concerning the Wonders of the World*), of ca. 1275; and the pseudonymous Marcus Graecus, *Liber ignium ad comburendos hostes* (*Book of Fire for the Burning of Enemies*), of ca. 1300. See A. R. Williams, “The Production of Saltpetre in the Middle Ages,” *Ambix* 22, no. 2 (1975): 125–33, 125. For their recipes, see J. R. Partington, *A History of Greek Fire and Gunpowder* (Cambridge: University of Cambridge, 1960), pp.42–90.

22. Herodotus, *Histories* 2.86–7; Pliny the Elder, *Natural History* 18.45.157, 19.26.84, 19.41.143, 30.10.29, and 31.46.106-22; Strabo, *Geography* 17.1.23; Proverbs 25:20; and Jeremiah 2:22. Many of these refer to “natron” (sodium carbonate), which was often translated as “niter.” See Cressy, *Saltpeter*, pp.12–13 (note 5), and Partington, *History of Greek Fire and Gunpowder*, pp.298–314 (note 21).

23. Medieval Gunpowder Research Group, “Making Saltpetre: Report 11,” 2013, p.5, <https://ahc.leeds.ac.uk/downloads/download/35/ho_medieval_gunpowder_research_group> (March 1, 2021). The chemical steps are: 1. Urea ((NH_2_)_2_CO) + bacteria (using the enzyme urease) → ammonia (NH_3_). 2. NH_3_ + *nitrosomonas* → NO_2–_ + H_2_O + H_+_. 3) NO_2–_ + *nitrobacter* → NO_3–_.

24. Robertson, “Reworking Seventeenth-Century Saltpetre,” 154 (note 5); Williams, “The Production of Saltpetre in the Middle Ages,” 131–3 (note 21).

25. Cressy, *Saltpeter*, pp.133–5 (note 5); Frey, “The Indian Saltpeter Trade,” 510–11 (note 7); Anthony Butler and John Moffett, “Saltpetre in Early and Medieval Chinese Medicine,” *Asian Medicine* 5, no. 1 (2009): 173–85; Sun Laichen, “Saltpeter Trade and Warfare in East Asia,” in Fujita Kayoka, Momoki Shiro, and Anthony Reid (eds.), *Offshore Asia: Maritime Interactions in East Asia before Steamships* (Cambridge: Cambridge University Press, 2013), pp.130–84.

26. Cressy, *Saltpeter*, p.130 (note 5).

27. Buchanan, “‘The Art and Mystery of Making Gunpowder,’” in Brett D. Steele and Tamera Dorland (eds.), *The Heirs of Archimedes*, pp.233–74 (note 9); Bengt Åhslund, “The Saltpetre Boilers of the Swedish Crown,” in Brenda J. Buchanan (ed.), *Gunpowder: The History of an International Technology* (Bath, UK: University of Bath Press, 1996), pp.163–81; Cressy, *Saltpeter*, pp.100, 104 (note 5). For sixteenth-century instructions for this process, see Georgius Agricola, *De Re Metallica*, 2nd ed., Herbert Clark Hoover and Lou Henry Hoover (eds.), trans. into English by Herbert Clark Hoover and Lou Henry Hoover (New York: Dover, 1950), pp.561–3, 561 n.9. The modern formula is Ca(NO_3_)_2_ + K_2_CO_3_ = CaCO_3_ + 2KNO_3_.

28. Cressy, *Saltpeter*, pp.42–54, 88–120, 166–73 (note 5).

29. Chase, *Firearms*, pp.58–65, 84–7 (note 20); Partington, *History of Greek Fire and Gunpowder*, pp.314–15 (note 21); Walter Panciera, “Saltpetre Production in the Republic of Venice from the Sixteenth to Eighteenth Century,” *ICON* 3 (1977): 155–66. Throughout the fifteenth century, nitraries were most common in Italy and Germany.

30. Cressy, *Saltpeter*, pp.42–5 (note 5).

31. Deborah Harkness, *The Jewel House: Elizabethan London and the Scientific Revolution* (New Haven: Yale University Press, 2007), pp.167–79.

32. Cressy, *Saltpeter*, pp.54–5, 78 (note 5). On these patents and grants, see, e.g., The National Archives, Kew, SP 12/16/30 fol. 59; Robert Lemon (ed.), *Calendar of State Papers, Domestic, Edward VI, Mary, and Elizabeth I (1547–1580)* (London: Public Records Office, 1856), Vol. 1, p.461, 511, 553; and Mary Anne Everett Green (ed.), *Calendar of State Papers Domestic, Elizabeth I (1581–1590)* (London: Her Majesty’s Stationery Office, 1865), Vol. 2, p.612.

33. Vannoccio Biringuccio, *De la Pirotechnia* (Venice: Ventorino Roffinello, 1540); Georgius Agricola, *De re metallica* (Basel: Hieronymus Froben and Nicholas Episcopius, 1556); Lazarus Ercker, *Beschreibung aller fürnemisten mineralischen Ertzt und Berckwercksarten* (Prague: Georg Schwartz, 1574); Whitehorne, *Certaine Wayes for the ordering of souldiours in battelray*. . . (note 2).

34. Newman, “From Alchemy to ‘Chymistry,’” p.515 (note 13).

35. Ibid. See also Ernst Darmstaedter, “Berg-, Probir- und Kunstbüchlein,” *Munchener Beitrage zur Geschichte und Literatur der Naturwissenschaften und Medizin* 2, no. 3 (1926): 101–206.

36. William Eamon, *Science and the Secrets of Nature: Books of Secrets in Medieval and Early Modern Culture* (Princeton: Princeton University Press, 1994), pp.112–34.

37. Pamela H. Smith, “Vermilion, Mercury, Blood, and Lizards: Matter and Meaning in Metalworking,” in Ursula Klein and Emma C. Spary (eds.), *Materials and Expertise in Early Modern Europe: Between Market and Laboratory* (Chicago: University of Chicago Press, 2010), pp.29–49. Smith quotes the anonymous *Goldsmith’s Storehouse* (ca. 1604), fol. 55*r.* Notably, this does not, in fact, produce saltpeter.

38. British Library, MS Sloane 1723, fols. 70*v*–93*r*. This appears to be a recipe for producing various metallic sulfates.

39. E.g., British Library, MS Sloane 1386, fols. 1*v*–2*r*, 5*v*, 6*v*, 9*r*–10*r*, 22*v*, 24*r–v*, and 29*v*–30*r*; Wellcome Library, MS 4755, fols. 277*r*–321*r*.

40. Marsilio Ficino, *De vita coelitus comparanda* (Florence: Antonio di Bartolomeo Miscomini, 1489); Giovanni Augurelli, *Chrysopoeia libri tres* (Basel: Johannes Froben, 1518); Heinrich Cornelius Agrippa, *De occulta philosophia libri tres* (Paris: C. Wechilus, 1531; Cologne: Johannes Soter, 1533). See Newman, “From Alchemy to ‘Chymistry,’” pp.497–517 (note 13).

41. Ercker, *Beschreibung aller fürnemisten mineralischen Ertzt und Berckwercksarten*, fol. 134.

42. Lawrence Principe, *The Secrets of Alchemy* (Chicago: University of Chicago Press, 2013), pp.35–7; William R. Newman, *The “Summa perfectionis” of Pseudo-Geber* (Leiden: Brill, 1991), esp. pp.143–92.

43. Paracelsus, *Sämtliche Werke*, Karl Sudhoff and Wilhelm Mattießen (eds.) (Hildesheim: Georg Olms Verlag, 1996), Vol. 11, p.349; Vol. 13, p.393. See also Bruce T. Moran, *Distilling Knowledge: Alchemy, Chemistry, and the Scientific Revolution* (Cambridge, MA: Harvard University Press, 2005), pp.72–3.

44. Newman, “From Alchemy to ‘Chymistry,’” pp.513–17 (note 13). These theories are derived from the chemist Georg Ernst Stahl, who wrote historically about alchemical theory in the 1710s and 1720s. The mercurial school remained the most common throughout the early modern period, but both the *sal nitrum* and vitriol schools (where metallic sulfates were the starting point for the philosopher’s stone) vied with it. See Georg Ernst Stahl, *The Philosophical Principles of Universal Chemistry*, trans. into English by Peter Shaw (London: J. Osborn and T. Longman, 1730), p.395. Recently, historians have begun to question these clear-cut divisions. See, e.g., Jennifer Rampling, *The Experimental Fire: Inventing English Alchemy, 1300–1700* (Chicago: University of Chicago Press, 2020).

45. Michael Sendivogius, *Novum lumen chemicum* (Frankfurt and Prague: 1604). This work was immensely popular. At least thirty editions appeared between 1604 and the end of the eighteenth century. The most well-known English edition was John French, *A New Light of Alchymie: Taken out of the Fountaine of Nature, and Manuall Experience, to which is Added a Treatise of Svlphvr* (London: Richard Coates for Thomas Willis at the Bible in Little Britain, 1650). I have quoted from this edition. On Sendivogius’s alchemy, see Zbigniew Szydło, “The Alchemy of Michael Sendivogius: His Central Nitre Theory,” *Ambix* 40, no. 3 (1993): 129–46; Zbigniew Szydło, “The Influence of the Central Nitre Theory of Michael Sendivogius on the Chemical Philosophy of the Seventeenth Century,” *Ambix* 43, no. 2 (1996): 80–97; Paolo Alves Porto, “Michael Sendivogius on Niter and the Preparation of the Philosopher’s Stone,” *Ambix* 48, no. 1 (2001): 1–16; Rafał T. Prinke, “New Light on the Alchemical Writings of Michael Sendivogius (1566–1636),” *Ambix* 63, no. 3 (2016): 217–43.

46. Thomas Leng, *Benjamin Worsley (1618–1677): Trade, Interest, and the Spirit in Revolutionary England* (Woodbridge, UK: Boydell Press, 2008), p.97; William R. Newman, *Gehennical Fire: The Lives of George Starkey* (Cambridge, MA: Harvard University Press, 1994), pp.87–90.

47. On *semina* theory, see, e.g., Jole Shackleford, “Seeds with a Mechanical Purpose: Severinus’s *Semina* and Seventeenth-Century Matter Theory,” in Allen G. Debus and Michael T. Walton (eds.), *Reading the Book of Nature: The Other Side of the Scientific Revolution* (St. Louis: Truman State University Press, 1998), pp.15–44; Hiro Hirai, *Le concept de semence dans les théories de la matière à la Renaissance: De Marsile Ficin à Pierre Gassendi* (Turnhout: Brepols, 2005); Christoph Lüthy, “Seeds Sprouting Everywhere,” *Annals of Science* 64 (2007): 411–20. Sendivogius uses “*sperma*” and “*semina*” interchangeably in *Novum lumen chemicum*; in the first published English translation, John French translated these as “seed,” “sperme,” or “semen.”

48. Principe, *Secrets of Alchemy*, p.128 (note 42).

49. French, *New Light of Alchymie*, pp.43–4.

50. Ibid., p.44.

51. On Duchesne’s matter theory, see Hiro Hirai, “Paracelsisme, néoplatonisme et médecine hermétique dans la théorie de la matière de Joseph Du Chesne à travers son *Ad veritatem hermeticae medicinae* (1604),” *Archives internationales d’histoire des sciences* 51, no. 1 (2001): 9–37.

52. Roos, *The Salt of the Earth*, pp.12–25 (note 14); Allen G. Debus, *The English Paracelsians* (London: Oldbourne, 1965), pp.86–97.

53. Josephus Quercetanus, *Ad vertitatem hermeticae mediecinae ex Hippocratis veterumque decretis ac therapeusi. . .* (Paris: Abraham Saugrain, 1604). I have quoted from the first English translation, Josephus Quercetanus, *The Practise of Chymicall, and Hermeticall Physicke, for the Preservation of Health*, trans. by Thomas Tymme, Minister (London: Thomas Creede, 1605), [sig.] B4*r*.

54. Ibid., [sig.] G2*v*.

55. This differed slightly from Sendivogius, who simply thought of saltpeter as the mature philosophical mercury that interacted with philosophical sulfur in the soil and air to produce its vital effects. Duchesne instead made all of these qualities intrinsic to the “aerial niter” or the vital force found in the air. See Allen G. Debus, “The Paracelsian Aerial Niter,” *Isis* 55, no. 1 (1964): 43–61.

56. Joseph Quercetanus, *Liber de priscorum philosophorum verae medicinae material. . ..* (n.p.: S. Gervasii, 1603), p.20, quoted in Debus, “Paracelsian Aerial Niter,” 54 (note 55).

57. Cheney Culpeper, Notes on Worsley’s *Discourse* in Hartlib’s Hand, July 10, 1654, HP 39/2/14A.

58. Ibid., 15A.

59. Ibid.

60. Cheney Culpeper to Benjamin Worsley, undated [likely 1648/9], HP 13/223A.

61. On Nuysement, see Wallace Kirsop, “Clovis Hesteau, sieur de Nuysement, et la littérature alchimique en France à la fin du XVIe et au début du XVIIe siècle” (Thèse de doctorat, Paris, Université de Paris, 1960).

62. Anonymous, “A Question Concerning Fertility,” [Quæstio de fertilitate Omnibus Naturæ scrutatoribus indefessis proposita], undated, HP 30/5/16A. “An per spermaticum vaporosum fumum, à Centro terræ ascendentem; an per Coeli influentias, an verò per solam Aquam imprægnatam, corruptam seu fermentatum [altered from fermentatam]? tandem an terra, ratione divinæ benedictionis, contineat infinitam virtutem, igneam seminalem, et prolificam, cujus Thesaursus nunquam exhauriri possit? Profectò Multiplicitas Opinionum doctissimorum Philosophorum (videlicet Aristotelis, Rupescissce. Sendivogij, Nortonij, Cartesij, Helmontij, Digbij, White, Glauberi) hanc quæstionem et concernentium, ostendit difficultatem Resolutionis.”

63. On Worsley’s involvement in saltpeter projects, see Leng, *Benjamin Worsley*, pp.95–117 (note 46); William R. Newman and Lawrence M. Principe, *Alchemy Tried in the Fire: Starkey, Boyle, and the Fate of Helmontian Chymistry* (Chicago: University of Chicago Press, 2005), pp.239–44; Clericuzio, “Plant and Soil Chemistry in Early Modern England,” 564–6 (note 10).

64. Leng, *Benjamin Worsley*, p.97 (note 46); Antonio Clericuzio, *Elements, Principles, and Corpuscles: A Study of Atomism and Chemistry in the Seventeenth Century* (Dordrecht: Kluwer, 2003), pp.35–47.

65. French, *New Light of Alchymie*, p.6. For Worsley’s use of these ideas, see Leng, *Benjamin Worsley*, p.100 (note 46).

66. Benjamin Worsley to ?Frederick Clodius, c. summer 1654, HP 42/1/26A. For more on the Sendivogian influence on Worsley in particular, see Leng, *Benjamin Worsley*, pp.95–102 (note 46).

67. Leng, *Benjamin Worsley*, pp.80–96 (note 46).

68. Ibid., pp.20–1.

69. Chang, “Alchemy as a Study of Life and Matter: Reconsidering the Place of Vitalism in Early Modern Chymistry,” 326 (note 12).

70. Leng, *Benjamin Worsley*, pp.37–41 (note 46).

71. Samuel Hartlib to Benjamin Worsley, May 16, 1654, HP 66/4/1B.

72. Ibid.

73. Benjamin Worsley, *De Nitro Theses Quædem*, HP 39/1/16A–20B.

74. Johann Rudolf Glauber, *The Works of the Famous and Highly Experienced Chemist, John Rudolph Glauber*, trans. into English by Christopher Packe (London: Thomas Milbourne, 1689), p.309. Originally published as Johann Rudolf Glauber, *Dess Teutschland Wohlfahrt* (Amsterdam: Jansson, 1656).

75. Benjamin Worsley, *De Nitro Theses Quædem*, HP 39/1/16A and 19B.

76. Hugh Plat, *Floraes Paradise* (London: H.L. for William Leake, 1608); Hugh Plat, *The Jewel House of Art and Nature* (London: Peter Short, 1594); Francis Bacon, *Sylva sylvarum, or a Natural History in Ten Centuries*. . . (London: W. Lee, 1627). On these experiments, see Clericuzio, “Plant and Soil Chemistry in Early Modern England,” 555–60 (note 10).

77. Cressy, *Saltpeter*, pp.90–2 (note 5).

78. Ibid., pp.133–5, 139.

79. Chaudhuri, *The English East India Company*, pp.189–90 (note 11).

80. Frey, “The Indian Saltpeter Trade,” 512–22 (note 7); Buchanan, “Saltpetre: A Commodity of Empire,” in Brenda Buchanan (ed.), *Gunpowder, Explosives, and the State*, pp.67–90, p.77 (note 9).

81. Webster, *The Great Instauration*, pp.324–60, 384–402, 465–83 (note 15); Paul Slack, *The Invention of Improvement: Information and Material Progress in Seventeenth-Century England* (Oxford: Oxford University Press, 2015), pp.1–12, 91–128; Paul Warde, “The Idea of Improvement, c. 1520–1700,” in Richard W. Hoyle (ed.), *Custom, Improvement, and the Landscape in Early Modern Britain* (Farnham, UK: Ashgate, 2011), pp.127–48. On cornucopianism, see Fredrik Albritton Jonsson, “The Origins of Cornucopianism: A Preliminary Genealogy,” *Critical Historical Studies* 1, no. 1 (2014): 151–68.

82. Carl Wennerlind, *Casualties of Credit: The English Financial Revolution, 1620–1720* (Cambridge, MA: Harvard University Press, 2011), pp.1–18, 61–83.

83. Benjamin Worsley and Johann Moriaen (?), “Notes on salpetre. . .,” May 18, 1653, HP 39/1/11B. See Newman and Principe, *Alchemy Tried in the Fire*, p.240 (note 63); Leng, *Benjamin Worsley*, p.99 (note 46).

84. Robert Boyle, *The Sceptical Chymist* (London, 1661), in Michael Hunter and Edward B. Davis (eds.), *The Works of Robert Boyle*, 14 vols., Vol. 2 (London: Pickering and Chatto, 1999–2000), p.351.

85. On the “keys” or “operations” to creating the philosopher’s stone, see Principe, *Secrets of Alchemy*, p.144 (note 42). On these operations as metaphors used in botany and plant chemistry, see Dana Jalobeanu and Oana Matei, “Treating Plants as Laboratories: A Chemical History of Vegetation in 17th-Century England,” *Centaurus* 62, no. 3 (2020): 542–61.

86. Samuel Hartlib, *Ephemerides 1650 Part I*, January–February, HP 28/1/39A–39B.

87. Ibid., 39B–40A. On alchemical secrecy, see Eamon, *Science and the Secrets of Nature* (note 36); Principe, *The Secrets of Alchemy*, esp. pp.137–72 (note 42); Tara Nummedal, *Alchemy and Authority in the Holy Roman Empire* (Chicago: University of Chicago Press, 2007), pp.119–46.

88. Peter Smith to John Beale, December 23, 1651, HP 67/23/3A–5A.

89. Ibid.

90. Samuel Hartlib, *Ephemerides of 1653* (March–May), HP 28/3/62A. Hartlib does not provide first names for these figures.

91. John T. Young, *Faith, Medical Alchemy, and Natural Philosophy: Johann Moriaen, Reformed Intelligencer, and the Hartlib Circle* (Aldershot, UK: Ashgate, 1998), pp.196–206.

92. Johann Moriaen, “Notes on Saltpeter in Scribal Hand G, ? & Johann Morian,” May 18, 1653, HP 39/1/11A. Internally, Hartlib retitled this with the simple heading “Observations on Saltpetre.”

93. Ibid., 11B.

94. Ibid., 11A.

95. On early modern alchemical definitions of fermentation, see Walter Pagel, *Joan Baptista Van Helmont: Reformer of Science and Medicine* (Cambridge: Cambridge University Press, 1982), pp.79–87; Joseph S. Fruton, *Fermentation: Vital or Chemical Process?* (Leiden: Brill, 2006), pp.8–39.

96. Moriaen, “Notes on Saltpeter in Scribal Hand G, ? & Johann Morian,” May 18, 1653, HP 39/1/11A.

97. Leng, *Benjamin Worsley*, pp.187–95 (note 46).

98. Michael Hunter, *Boyle: Between God and Science* (New Haven: Yale University Press, 2009), pp.75–9; Newman and Principe, *Alchemy Tried in the Fire*, p.215, 215 n.26 (note 63).

99. Samuel Hartlib, *Ephemerides of 1654* (January–April), HP 29/4/9A; Hartlib to Boyle, April 9 and May 17, 1659, in *The Works of the Honourable Robert Boyle*, Vol. 6, pp.116–17, 118, and 125; Oldenburg to Hartlib, April 30, 1659, HP 39/3/22B.

100. Reginald Lennard, “English Agriculture under Charles II: The Evidence of the Royal Society’s ‘Enquiries,’” *Economic History Review* 4, no. 1 (1932): 23–45; Michael Hunter, *Establishing the New Science: The Experience of the Early Royal Society* (Woodbridge, UK: Boydell, 1989), pp.73–122; Clericuzio, “Plant and Soil Chemistry in Early Modern England,” 582–3 (note 10).

101. Robertson, “Reworking Seventeenth-Century Saltpetre,” 150–2 (note 5); Leng, *Benjamin Worsley*, pp.18–25 (note 46).

102. Smith and Secoy, “A Compendium of Inorganic Substances Used in European Pest Control before 1850,” 1184 (note 10); Smith and Secoy, “Salt as a Pesticide, Manure, and Seed Steep,” 514 (note 10).

103. G. E. Fussell, *The Classical Tradition in West European Farming* (Teaneck, NJ: Farleigh Dickinson University Press, 1972).

104. Hugh Plat, “Diverse New Sorts of Soyle Not Yet Brought into Any Publique Use,” in *Jewel House of Art and Nature*, p.3 [separate pagination] (Note 76).

105. Smith and Secoy, “A Compendium of Inorganic Substances Used in European Pest Control before 1850,” 1184 (note 10); Clericuzio, “Plant and Soil Chemistry in Early Modern England,” 555–7 (note 10); Ayesha Mukherjee, “*In Vulcano Veritas*: Sir Hugh Platt’s Alchemical Exchanges,” in Subha Mukerji, Dunstan Roberts, Rebecca Tomlin, and George Oppitz-Trotman (eds.), *Economies of Literature and Knowledge in Early Modern Europe: Change and Exchange* (London: Palgrave Macmillan, 2020), pp.207–37, 216–19. Notably, this procedure existed in the ancient Roman world. See Virgil *Georgics* 1.10; Pliny the Elder *Natural History* 18.45.

106. Francis Bacon, *Sylva sylvarum, or a Natural History in Ten Centuries*. . . (London: W. Lee, 1627), p.117.

107. Samuel Hartlib, *Notes on Saltpeter*, February 6, 1652, HP 39/1/4A. “Vtilissimum Generi Humano foret illud multiplicandi frumenti Artificium. Non dubito rem esse possibilem, et vegetari posse semen per Lixivium quædam corroborantia in quibus conficiendis principatus.” In modern chemical terms, this lixivium is sodium hydroxide (NaOH), also known as lye and caustic soda.

108. Cressy Dymock, “A Discoverie for Division Or Setting Out of Land,” Cressy Dymock et al., Part 2 (London: Richard Wodenotche, 1653), p.13.

109. Gabriel Plattes, “A Treatise. . .Containing Certain Notes, Observations, Experiences and Improvements of Husbandry, with the Judgement upon Them of an Experiences Husbandman, Who Hath Also Brought the Invention of Setting Corn to Greater Perfection,” in Samuel Hartlib, *His Legacy of Husbandry* (London: J.M. for Richard Wodenotche, 1651), p.192.

110. John Worlidge, *Systema Agriculturæ* (London: T. Johnson for Samuel Speed, 1668), p.56. Emphases in original. Worlidge quoted Virgil as saying, “Semina vidi equidem multos medicare serentes et nitro prius—profundere—,” which comes from Virgil *Georgics* 1.193–5. The full text reads: “Semina vidi equidem multos medicare serentis et nitro prius et nigra perfundere amurca, grandior ut fetus siliquis fallacibus esset” [“I myself have seen many sowers steep their pulse seeds, soaking them with niter and black oil-lees, so that the fruit may swell within the deceitful pods”]. Henry Rushton Fairclough (ed.), *Virgil: Georgics: Aeneid I-VI: Eclogues*, trans. Henry Rushton Fairclough (Cambridge, MA: Harvard University Press, 1978), pp.94–5. By “niter,” Virgil likely meant sodium carbonate rather than potassium nitrate.

111. Ibid. Emphases in original.

112. On this alchemical terminology, see William Newman, “Alchemical Glossary,” <http://webapp1.dlib.indiana.edu/newton/reference/glossary.do> (July 10, 2018). Due to the general terminology for many substances, it is difficult to tell what Worlidge meant by “alcali.” Alkali referred to several basic materials, and in the context of calcination could produce a number of oxide, hydroxide, and carbonate compounds. A “vegetable alkali” usually meant potassium carbonate (K_2_CO_3_), which was found in wood ash and was the last chemical product before nitrates replaced carbonates in the artificial formation of saltpeter. On “fixed salt” (likely equivalent to the “vegetable alkali”), see Kathleen Ahonen, “Johann Rudolf Glauber: A Study of Animism in Seventeenth-Century Chemistry” (PhD diss., University of Michigan, 1972), p.107 n.59.

113. Walter Blith, *The English Improver Improved* (London: John Wright, 1652), p.127; and Adolphus Speed, *Adam out of Eden* (London: Henry Brome, 1659), p.105.

114. Charles Cotton, *The Planters Manual* (London: Henry Brome, 1675), p.88.

115. John Mortimer, *The Whole Art of Husbandry* (Dublin: George Grierson, 1721), pp.40, 41, 84, 194.

116. Jethro Tull, *The Horse-Hoing Husbandry*, 2nd ed. (London: A. Millar, 1743), p.66.

117. Thomas Hale, *A Compleat Body of Husbandry* (London: Osborn & Shipton, 1756), p.353. Copperas was the term for the crystallized form of green vitriol, or ferrous sulfate (FeSO_4_), also called *sal martis*.

118. Matthew Peters, *Winter Riches, or a Miscellany of Rudiments, Directions and Observations, Necessary for the Laborious Farmer, on a New Vegetable System of Agriculture. . .* (London: W. Flexney, 1771), p.133.

119. Smith and Secoy, “A Compendium of Inorganic Substances Used in European Pest Control before 1850,” 1184, 1186 (note 10).

120. F. A. Buttress and R. W. G. Dennis, “The Early History of Cereal Seed Treatment in England,” *Agricultural History Review* 21, no. 2 (1947): 93–103.

121. Buchanan, “‘The Art and Mystery of Making Gunpowder,’” in Brett D. Steele and Tamera Dorland (eds.), *The Heirs of Archimedes*, p.233 (note 9).

122. Cressy, *Saltpeter*, pp.42–54 (note 5).

123. Guldner et al., “From Farm to Gun and No Way Back,” 157–60 (note 10);” “Thomas Kaiserfeld, “Chemistry in the War Machine: Saltpeter Production in Eighteenth-Century Sweden,” in Brett D. Steele and Tamera Dorland (eds.), *The Heirs of Archimedes*, pp.275–92 (note 9)”

124. Cressy, *Saltpeter*, pp.72, 138 (note 5).

125. E.g., Simron Jit Singh, Helmut Haberl, Marian Chertow, Michael Mirtl, and Martin Schmid (eds.), *Long Term Socio-Ecological Research: Studies in Society – Nature Interactions across Spatial and Temporal Scales* (Dordrecht: Springer, 2013).

126. Nummedal, *Alchemy and Authority in the Holy Roman Empire*, pp.73–95 (note 87); Tara Nummedal, “Words and Works in the History of Alchemy,” *Isis* 102, no. 2 (2011): 330–7.

127. Vera Keller, *Knowledge and the Public Interest, 1575–1725* (Cambridge: Cambridge University Press, 2015).

